# Supplementary material for: Hyperuricemia Is an Early and Relatively Common Feature in Children with HNF1B Nephropathy but Its Utility as a Predictor of the Disease Is Limited
Source: J Clin Med. 2021 Jul 24;10(15):3265. doi: 10.3390/jcm10153265 (PMC8346958; doi:10.3390/jcm10153265)
Supplement: Supplementary file 1 [file jcm-10-03265-s001.zip › jcm-1260931-SI.pdf]

Supplementary

Table S1. Results of univariate regression analysis performed in the entire cohort of patients

Parameter	B	SE	Beta	t	F	R^2
**Age**	0.17	0.02	0.58	7.83***	61.31***	0.33
**Mutation**	0.71	0.28	0.23	2.56*	6.53*	0.05
**eGFR**	-0.02	0.00	-0.53	-6.29***	39.55***	0.28
**sMg**	-5.60	1.18	-0.41	-4.76***	22.66***	0.17
**Hypomagnesemia**	0.86	0.28	0.27	3.02**	9.12**	0.07
**IFG/DM**	0.19	0.39	0.41	4.92***	24.23***	0.17
**PTH**	0.02	0.00	0.42	4.07***	16.57***	0.18
**Hyperparathyroidism**	1.04	0.44	0.26	2.36*	5.57*	0.07
**FEMg**	0.21	0.05	0.41	4.48***	20.10***	0.17
**FEUA**	-0.18	0.06	-0.30	-3.08**	9.48**	0.09
**CKD**	0.98	0.29	0.43	3.35**	19.27**	0.18
**Hypertension**	1.196	0.35	0.29	3.41**	11.63**	0.09

*$p < 0.05$; **$p < 0.01$; ***$p < 0.001$. CKD, chronic kidney disease; DM, diabetes mellitus; eGFR, estimated glomerular filtration rate; FEMg, fractional excretion of Mg^{2+}; FEUA, fractional excretion of uric acid; IFG, impaired fasting glucose; PTH, parathyroid hormone; sMg, serum Mg^{2+}
